# Analysis of cartilage loading and injury correlation in knee varus deformity

**DOI:** 10.1097/MD.0000000000038065

**Published:** 2024-05-10

**Authors:** Hongjie Zhang, Jianxiong Ma, Aixian Tian, Bin lu, Haohao Bai, Jing Dai, Yanfei Wu, Jiahui Chen, Wei Luo, Xinlong Ma

**Affiliations:** a Tianjin University of Traditional Chinese Medicine, Tianjin, PR China; b Tianjin University Tianjin Hospital, Tianjin, PR China; c Tianjin Orthopedic Research Institute, Tianjin, PR China; d Kunming Medical University Affiliated Dehong Hospital/Dehongzhou People’s Hospital, Mangshi, China; e Tianjin Medical University, Tianjin, PR China.

**Keywords:** cartilage damage, finite element analysis, knee osteoarthritis, knee varus, stress distribution, Von-Mises stress

## Abstract

Knee varus (KV) deformity leads to abnormal forces in the different compartments of the joint cavity and abnormal mechanical loading thus leading to knee osteoarthritis (KOA). This study used computer-aided design to create 3-dimensional simulation models of KOA with varying varus angles to analyze stress distribution within the knee joint cavity using finite element analysis for different varus KOA models and to compare intra-articular loads among these models. Additionally, we developed a cartilage loading model of static KV deformity to correlate with dynamic clinical cases of cartilage injury. Different KV angle models were accurately simulated with computer-aided design, and the KV angles were divided into (0°, 3°, 6°, 9°, 12°, 15°, and 18°) 7 knee models, and then processed with finite element software, and the Von-Mises stress distribution and peak values of the cartilage of the femoral condyles, medial tibial plateau, and lateral plateau were obtained by simulating the human body weight in axial loading while performing the static extension position. Finally, intraoperative endoscopy visualization of cartilage injuries in clinical cases corresponding to KV deformity subgroups was combined to find cartilage loading and injury correlations. With increasing varus angle, there was a significant increase in lower limb mechanical axial inward excursion and peak Von-Mises stress in the medial interstitial compartment. Analysis of patients’ clinical data demonstrated a significant correlation between varus deformity angle and cartilage damage in the knee, medial plateau, and patellofemoral intercompartment. Larger varus deformity angles could be associated with higher medial cartilage stress loads and increased cartilage damage in the corresponding peak stress area. When the varus angle exceeds 6°, there is an increased risk of cartilage damage, emphasizing the importance of early surgical correction to prevent further deformity and restore knee function.

## 1. Introduction

Knee osteoarthritis (KOA) is a chronic, progressive condition that can lead to joint dysfunction in over 30% of individuals aged over 60.^[1,2]^ Its primary clinical manifestations include knee pain, swelling, and deformity.^[3]^ According to a 2010 study, the global age-standardized prevalence of KOA is 3.8%, and this prevalence significantly rises with age.^[4]^ Besides age and obesity, abnormal mechanical factors are pivotal in osteoarthritis pathogenesis.^[5]^ KOA primarily presents as a 3-dimensional lower limb deformity, particularly in the coronal plane, resulting in varus or eversion deformities.^[6]^ These deformities affect the load-bearing capacity of both the medial and lateral knee joint compartments, increasing pressure on cartilage and subchondral bone and ultimately worsening osteoarthritis progression.

Knee varus (KV) deformity is a significant contributor to KOA in adults,^[7]^ and its pathogenesis has been closely linked to increased cartilage loading within the medial tibiofemoral compartment, which accelerates the degeneration of medial compartment cartilage.^[8,9]^ This deformity can arise from tibial deformities, tibiofemoral and femoral syndesmosis or ligamentous structural abnormalities. Medial KOA develops when the medial compartment sustains damage due to an alteration in the lower extremity line of force, resulting in excessive medial load that surpasses its tolerance range.^[10]^ Alterations in the deformity angle and increased stress on cartilage can lead to cartilage damage, and when these changes surpass the joint’s compensatory capacity, it can result in cartilage damage and the progression of KOA.^[11]^ While there are numerous investigations on KV deformity leading to KOA,^[12]^ few studies have investigated the relationship between deformity magnitude and the distribution of force loads on femoral and tibial cartilage surfaces concerning the degree of cartilage damage. Further studies are warranted to explore the specific deformity angles associated with the loss of compensatory capacity in the knee joint to provide clinical guidance for precise treatment strategies.

Using traditional X-ray for assessing the KV angle often leads to non-accurate measurements,^[13]^ particularly when the patient’s positioning is nonstandard. This may result in issues like femur rotation and incomplete visualization of the femoral head, leading to inaccurate femoral head alignment, imprecise mechanical axes of the lower extremities, and a tendency for X-rays to miss deformities in the coronal and axial planes,^[14]^ thus compromising KV angle measurement. Computer-aided design (CAD) uses specialized software to present the image on a multidimensional coordinate system, which then allows the image model to be manipulated in 3 dimensions and permits it to be viewed from a variety of angles.^[15]^ With the rapid development of image-based CAD, CAD applications have become prevalent in diagnostic and preoperative surgical planning as they can enable more precise measurements of lower limb force lines and angles, thereby enhancing measurement accuracy.^[16]^

Although numerous accomplishments have been achieved in finite element analysis in regard to normal knee joints,^[17–19]^ few studies have been performed on finite element modeling using actual clinical patient injuries. To fill this gap, we designed this present study to: create 3-dimensional simulation models of KOA with varying varus angles using CAD; investigate stress distribution in the knee joint cavity and compare intra-articular loads among different models of varus KOA; and present the results of finite element analysis with correlation analyses of cartilage damage in clinical cases of KV.

## 2. Methods

### 2.1. Finite element analysis

#### 2.1.1. Data acquisition

**Figure 1.**
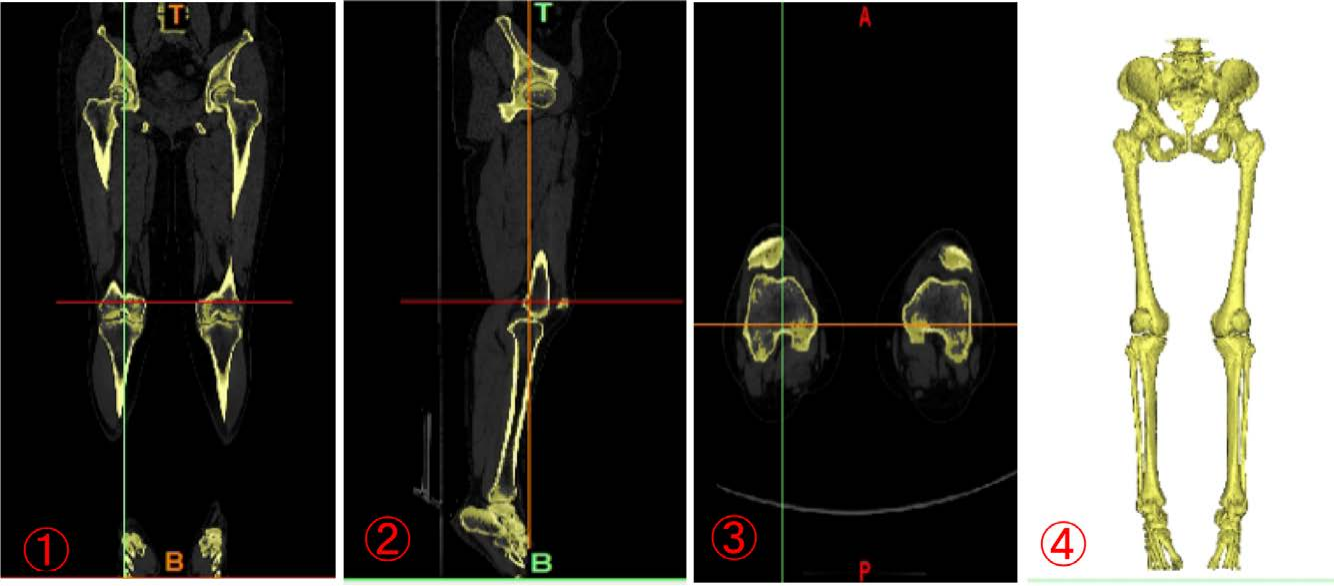
3D CT reconstruction. ①\②\③\④ CT images of both lower limbs in sagittal, coronal, cross-sectional, and 3D processing model, respectively.

A female patient aged 60, weighing 70 kg, with mild osteoarthritis and a normal force line, was selected for a knee joint CT scan at Tianjin Hospital (Tianjin City, China) using the Lightspeed VCT scanner (GE, Las Vegas, NV, USA) with a layer thickness of 0.625 mm. The right knee joint of the patient was used as the modeling subject. The Digital Imaging and Communications in Medicine (DICOM) file of the right knee joint, obtained from the CT scan, was imported into the Mimics21 software. Using different CT threshold scanning images, the segmentation threshold was set to 142–1805 Hounsfield units (HU) through threshold segmentation. Initially, the cortical bone masks of the femur and tibiofibula were extracted using the region growth tool. The edited bone masks were further segmented using the “add” and “erase” functions in the Edit Mask tool to create separate masks for the femur, tibiofibula, and patella. The outer contour of each structure was refined and closed, and any cavities were filled. Subsequently, the edited bone masks were geometrically reconstructed in 3D using the Calculate 3D Model function to create a 3D bone model, which was then outputted in STL format (Fig. 1).

#### 2.1.2. Construction of the 3D solid model of each structure

**Figure 2.**
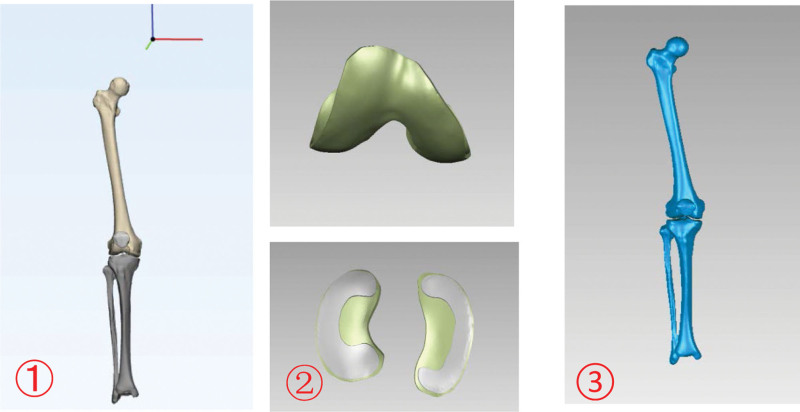
① 3D bone model after 3-MATIC optimization. ② Design-generated models of femoral condylar cartilage, tibial cartilage and meniscus. ③ The materialization model.

The STL file of the right lower limb bone model was imported into 3-MATIC for further refinement and mesh optimization to achieve a high-quality geometric model. Next, the 3D geometric model of each component of the knee joint was imported into the Geomagic Studio software for additional refinement. Soft tissue structures such as the femoral cartilage, tibial cartilage, and meniscus were delineated based on human anatomy definitions, and their characteristics were aligned with the bone structures’ surfaces. These structures were then constructed and fitted onto the surfaces using the Accurate Surface Module to create 3D solid models for each component (Fig. 2). Lastly, the final models were exported in STEP format.

#### 2.1.3. Modeling KV from different angles

**Figure 3.**
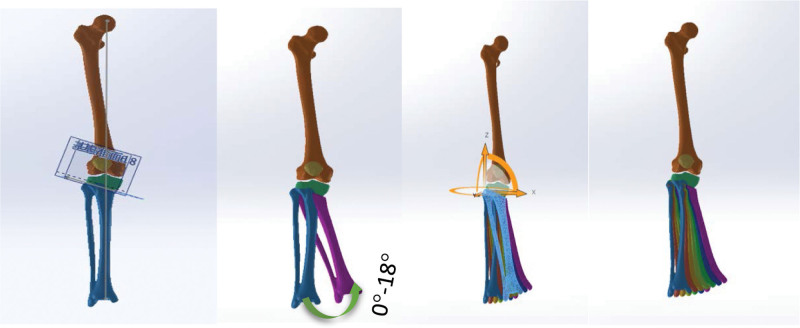
0° to 18° varus with a high tibial osteotomy hinge of the knee joint.

All the 3D solid model data of the knee joint were imported into the SOLIDWORKS software to assemble each structure of the knee joint and create the comprehensive knee joint model, which was then used as a foundation to model various varus angle scenarios, such as simulating high tibial osteotomy (HTO) surgery for osteotomy. The varus tibia models were constructed around the osteotomy hinge in the sagittal plane, establishing the standard 0° position, after which 3D models for varus angles of 3°, 6°, 9°, 12°, 15°, and 18° were created (Fig. 3).

#### 2.1.4. Mesh modeling

**Figure 4.**
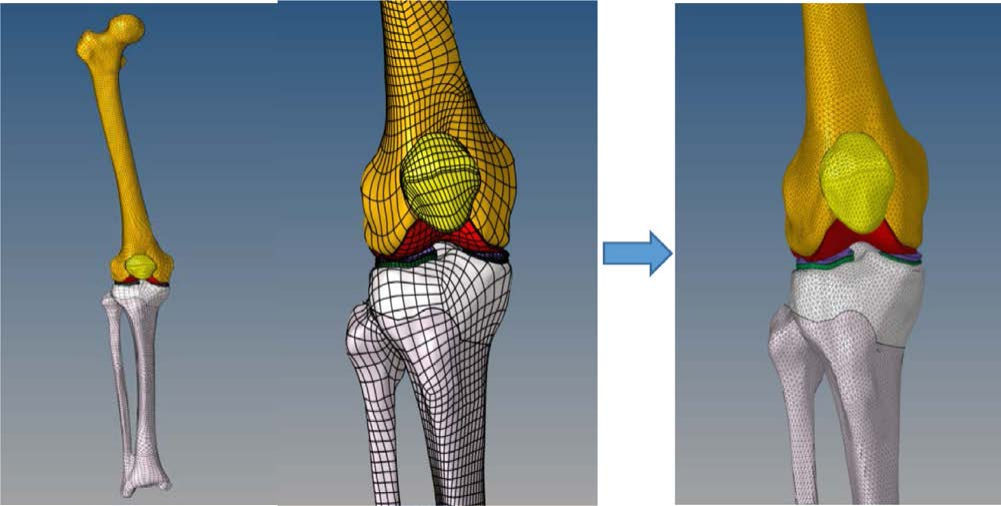
Bone and cartilage mesh modeling.

The STEP files of the 7 sets of models were imported into HyperMesh 13.0 (Altair, Frisco, TX, USA) for meshing. Mesh partitioning was conducted based on a trade-off between computational accuracy and computational cost. Subdividing the mesh can enhance computational accuracy but also prolong computational time. Additionally, hexahedral meshes generally offer better computational accuracy and shorter computation times compared to tetrahedral meshes. However, hexahedral meshing demands more complex operations and may not always be feasible for intricate models. Thus, in this experiment, we focused on investigating biomechanical changes in the knee joint. Therefore, for the bone model, which has minimal influence on the internal contact analysis results of the knee joint, a tetrahedral meshing approach was adopted with a cell size set at 2 mm, as described in the previous literature.^[13]^ Conversely, since the biomechanical alterations in cartilage are of primary interest in this study, mesh refinement was applied to the cartilage, meniscus, ligaments, and other soft tissue structures, with a grid cell size set at 1 mm^[20]^ (Fig. 4), the mesh size passed the convergeny test.

#### 2.1.5. Tissue modulus of elasticity relationship

The mesh model was exported as an INP file and imported into Abaqus 2021 for stress distribution calculations. Then, the femur, fibula, and tibia were characterized as isotropic linearly elastic materials.^[21]^ Similarly, the cartilage and menisci were also described as linear elastic and isotropic materials.^[22]^ Material properties were assigned as per Table 1 and then assembled accordingly. For the ligament structure between the femur and tibia, it was substituted with linear spring elements. Each ligament comprised 2 springs, each with a stiffness of 800 N/mm.^[23]^

**Table 1:** Tissue modulus of elasticity.

	Young modulus (MPa)	Poisson ratio
Bones^[19]^	20,000	0.3
Gristle^[19]^	5	0.46
Meniscus^[23]^	59	0.49

MPa = megapascals.

#### 2.1.6. Boundary conditions and loads

The boundary conditions and loads were established as follows: the distal ends of the fibula and tibia were fixed in all degrees of freedom, while the femur remained free in all degrees of freedom^[24]^; then, the contact surface properties between the cartilage of the femur and tibia were defined as frictionless face-to-face contact with finite slip. The contact pressure-gap relationship between the surfaces was set to a “hard” contact using a penalty function algorithm. Contacts involving the meniscus and ligaments were treated as binding constraints to model the connection within the knee joint.^[25]^

**Figure 5.**
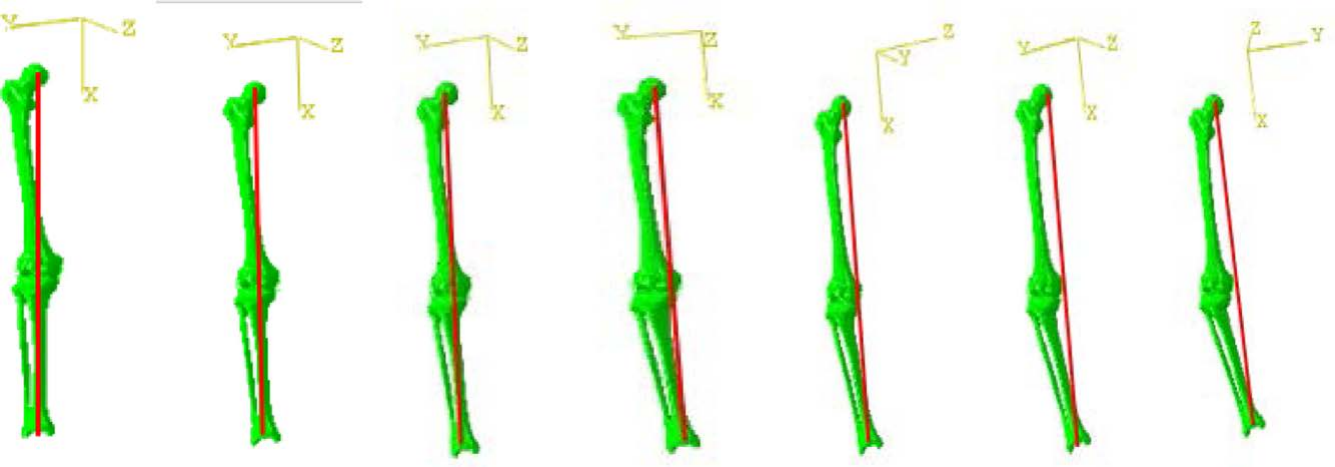
0°, 3°, 6°, 9°, 12°, 15°, and 18°KV finite element models. KV = knee varus.

**Figure 6.**
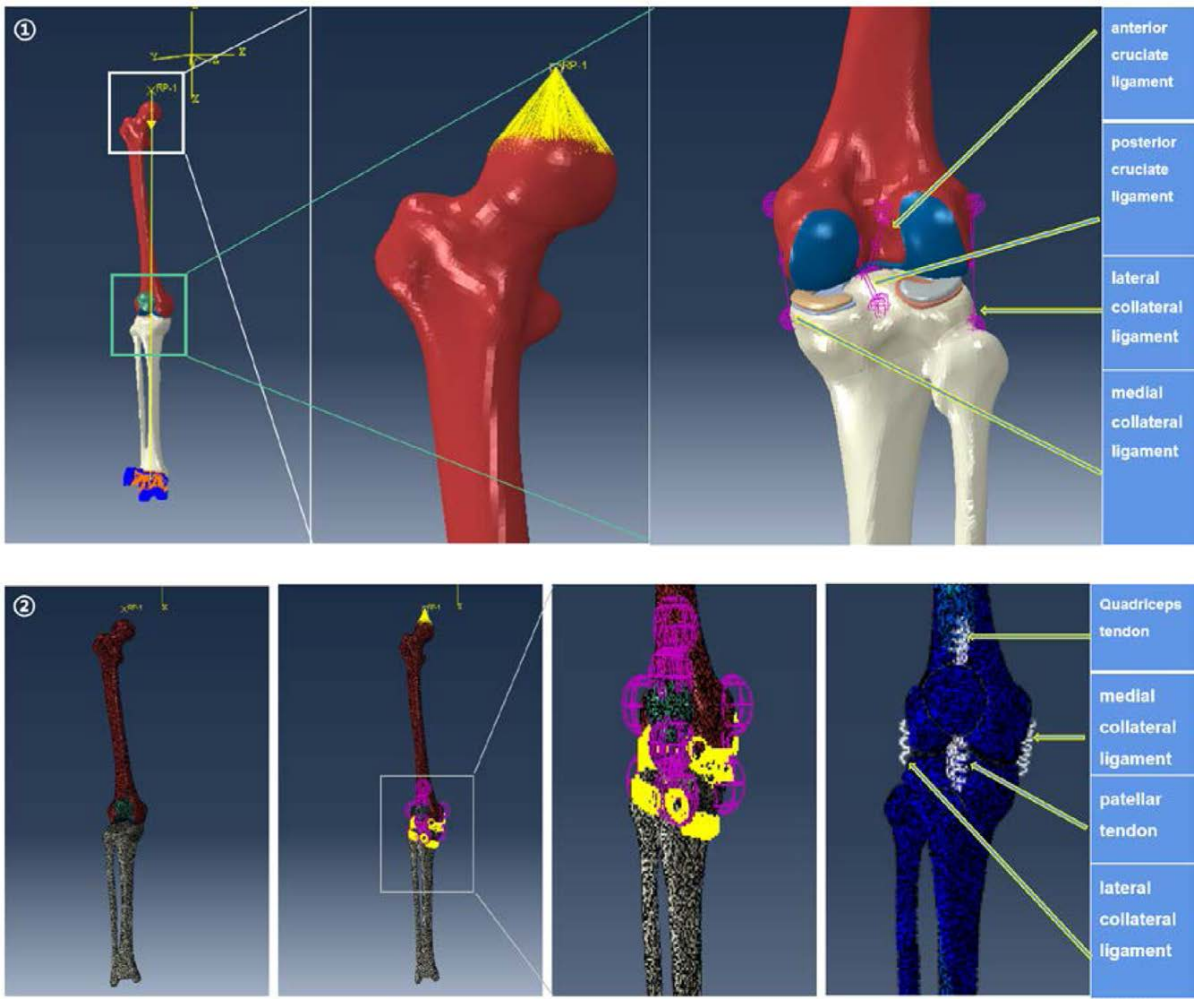
Lower extremity stress load models.

In this study, we conducted a static finite element analysis to simulate a scenario where the patient stood on one foot, and different angle finite element models were established (Fig. 5). To accurately replicate lower limb force loading, we implemented a local coordinate system for the right lower limb in the preprocessing module of ABAQUS 2021 software. The origin was placed at the center of the femoral head, and the *X* axis was aligned along the line connecting the femoral head center to the distal tibia, representing the lower limb’s mechanical shaft. A reference point for stress loading was set 10 mm above the proximal surface of the femoral head. A concentrated force of 740 N was applied to the femoral reference point,^[13]^ directed along the mechanical axis (*X* axis) of the right lower limb, and these loading boundary conditions were made consistent across all models (Fig. 6). Lastly, we calculated the distribution of Von-Mises stress (VMS) values for femoral and tibial cartilage at various deformity angles, focusing on the peak VMS.

#### 2.1.7. Parametric analysis

**Figure 7.**
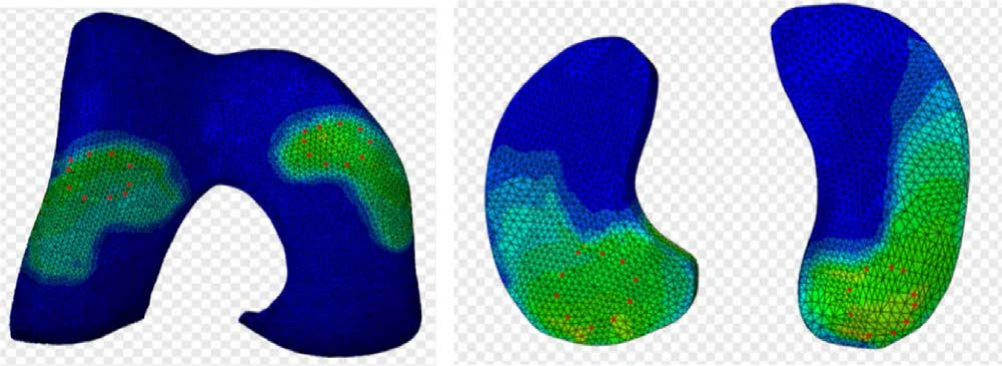
The 10 points of cartilage contact surface of femoral condyles and medial–lateral tibial plateau.

Parametric analysis was conducted by recording the distribution of peak VMS values for both femoral and tibial cartilage, and these values were regarded as indicators of the risk of cartilage damage. Specifically, 10 points were selected at the center of each cartilage contact surface, with a radius of 3 mm. The average VMS was calculated at these points to provide further assessment of the change in stress values (Fig. 7).

## 3. Clinical information and methods

### 3.1. Clinical information

Study participants: between March and August 2023, with the approval of the Ethics Committee of Tianjin Hospital, a total of 203 patients were recruited from Tianjin Hospital digital orthopedics. The patients had a mean age of 59.30 years and comprised 70 males and 133 females. They came to our hospital due to knee pain and were categorized into 7 groups based on their preoperative varus angle measurements (<3°, <6°, <9°, <12°, <15°, <18°, and ≥18°). All patients experienced clinical symptoms of medial or anteromedial knee pain and exhibited varying degrees of proximal tibial varus deformity. However, their pain was unresponsive to conservative treatment, significantly impacting their daily lives and work. All patients and their families provided informed consent for knee-conserving surgical treatment.

The study inclusion criteria were as follows: underwent comprehensive preoperative imaging assessments, including knee CT scans with thin-layer scanning and 3D reconstruction and MRI examinations, covering the full length of both lower limbs in a weight-bearing position, as well as weight-bearing anterior and lateral positions of the knee joint and patellar axial positioning; underwent preoperative laboratory tests, including biochemistry evaluations; and were eligible for surgical treatment.

The exclusion criteria were as follows: patients with bone-related conditions such as knee joint infection, rheumatoid arthritis, gout, hemophilic arthritis, or bone tumors; individuals with varus deformity exceeding 25° or flexion contracture >15°; patients with limb hemiparesis resulting from cerebral hemorrhage, cerebral infarction sequelae, or those exhibiting poor muscle strength in the lower limbs; patients with compromised cardiorespiratory function or multiple underlying diseases that rendered them unable to tolerate surgery; and individuals who demonstrated ineffective cooperation with functional exercises, poor compliance, or a history of missing scheduled follow-up appointments were all excluded from the study.

### 3.2. Methodology

#### 3.2.1. Clinical patient varus angle measurement

**Figure 8.**
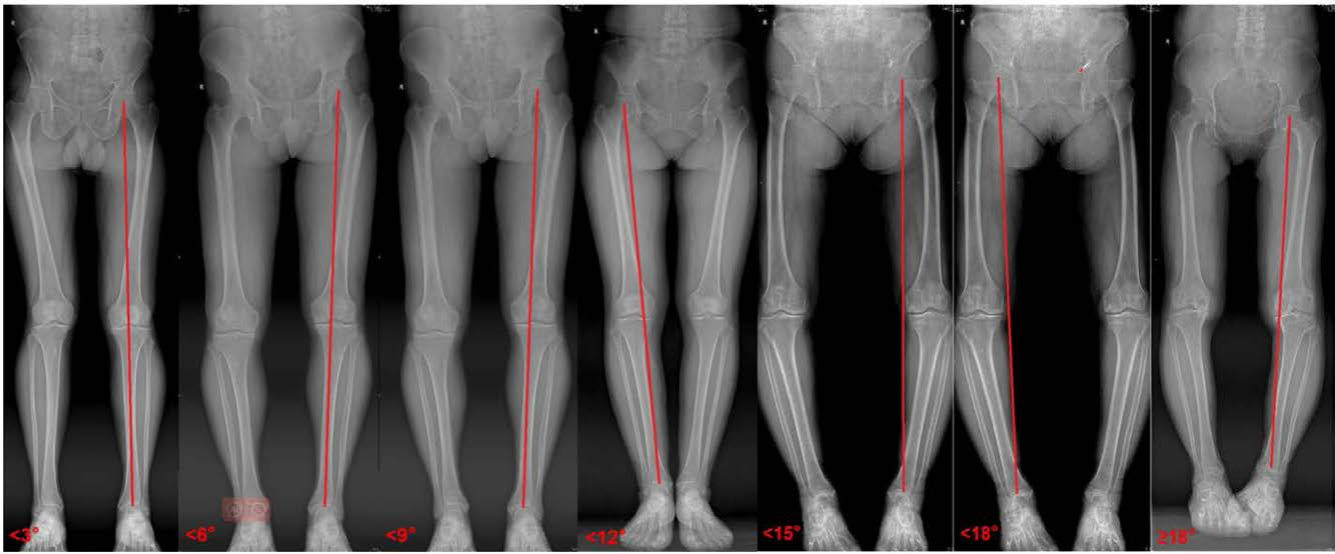
X-ray performance of 7 interval groupings (<3°, <6°, <9°, <12°, <15°, <18°, and ≥18°).

CT and X-ray data for each patient were input into computer software, and the varus angle of each patient’s lower limbs was individually measured using CAD and recorded. These measurements were then grouped into 7 intervals: <3°, <6°, <9°, <12°, <15°, <18°, and ≥18°, which corresponded to the respective finite element model angles of 0°, 3°, 6°, 9°, 12°, 15°, and 18° as seen in X-ray images (Fig. 8).

#### 3.2.2. Grading of cartilage damage

A total of 31 patients with deformity angles <3° (mean age 42.76 ± 18.75 years, comprising 13 males and 18 females) underwent arthroscopic cleanup and repair of tissue structures within the joint cavity. Intraoperative microscopic examinations were performed to assess damage in the medial, patellofemoral, and lateral compartments of the knee. On the other hand, 172 patients with deformity angles <6°, <9°, <12°, <15°, <18°, and ≥18° (mean age 62.68 ± 6.91 years, with 55 males and 117 females) underwent HTO in combination with arthroscopic cleanup and repair, utilizing a customized guide plate from the Tianjin Orthopaedic Research Institute for preoperative planning. Intraoperative microscopic evaluations were also conducted to assess and document damage in the knee’s medial interphalangeal, patellofemoral, and lateral interphalangeal compartments.

**Figure 9.**
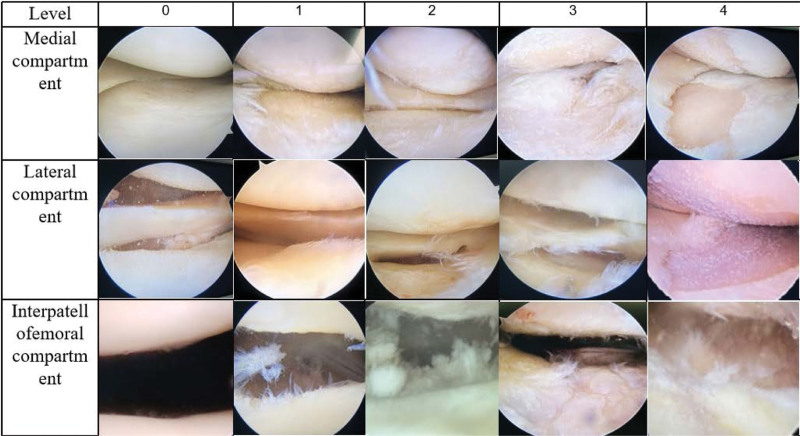
Reference for grading cartilage damage in different compartments.

**Figure 10.**
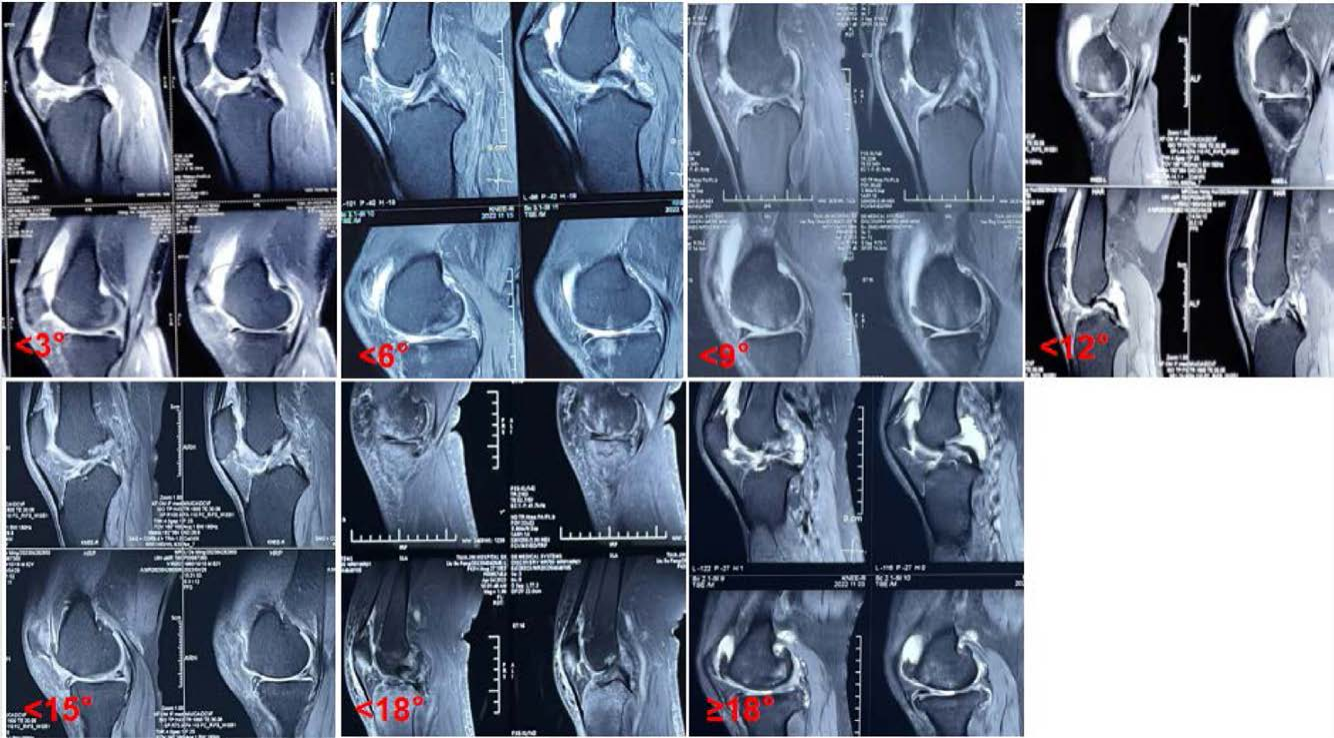
MRI performance in 7 interval groupings (<3°, <6°, <9°, <12°, <15°, <18°, and ≥18°).

Cartilage grading in the medial compartment, patellofemoral compartment, and lateral compartment of the knee was performed in accordance with the arthroscopic grading of joint lesions as scored by the International Society for Cartilage Repair (Table 2),^[26]^ with reference to intraoperative arthroscopic grading (Fig. 9). The results of microscopic damage grading for each group were combined with clinical MRI data (Fig. 10) to ensure a more accurate assessment of damage. Since most patients with varus deformity exhibited grade 4 cartilage damage in the medial and patellofemoral compartments, evaluating and distinguishing the overall joint injury severity posed a challenge. To address this, the study employed a cumulative approach, considering the degree of injury in each joint compartment. Specifically, the grading was as follows: grade 0 when there was no cartilage damage in any of the 3 compartments; grade 1 for 3 intercompartmental injuries, with none reaching grade 4; grade 2 for 1 intercompartmental injury at grade 4; grade 3 for 2 intercompartmental injuries at grade 4; and grade 4 for 3 intercompartmental injuries at grade 4.

**Table 2:** Arthroscopic grading of the articular lesions based on International Cartilage Repair Society Score.^[26]^

Grade	Description
0	Normal intact cartilage
1	Chondral softening and blistering, superficial lesions, fissures and cracks, soft indentation
2	Fraying, lesions, and fissures extending down to <50% of cartilage depth
3	Partial loss of cartilage thickness, cartilage lesions extending down >50% of cartilage depth as well as down to the calcified layer
4	Full-thickness cartilage loss with exposure of the subchondral bone

### 3.3. Statistical analysis

The finite element method was used to compare load distribution among different varus models and assess peak VMS values in each compartment. Correlation analysis was conducted with clinical cartilage injury statistics. Statistical analysis using 1-way ANOVA was performed using the SPSS 27.0.1 software. *P* < .01 was considered statistically significant, and the data were visualized using GraphPad Prism 8.0.1.

## 4. Results

### 4.1. Finite element analysis results

**Figure 11.**
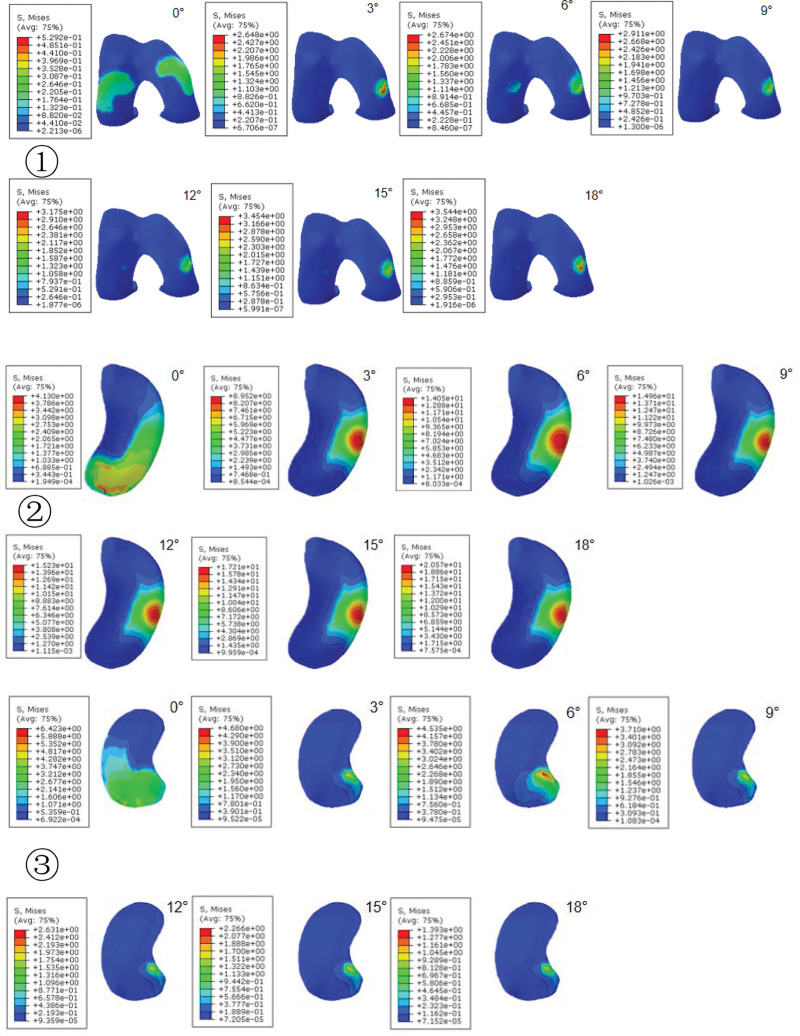
Finite element analysis comparing different KV model load zones and VMS values. ① Femoral condylar load zone and VMS value distribution; ② medial tibial plateau load zone and VMS value distribution, and; ③ lateral tibial plateau load zone and VMS value distribution. KV = knee varus, VMS = Von-Mises stress.

As the varus angle increased, we observed a corresponding inward shift of the lower limb mechanical axis. Under the consistent 740 N compression load, the VMS in different compartments of the knee joint exhibited gradual changes (Figures 11 and 12①). For instance, at 0°, the medial and lateral femoral condyles experienced the largest force distribution area, which gradually decreased from 3° to 18°, and the VMS peak in the medial femoral condyles exhibited a slight upward trend, ranging from 2.64 to 3.54 megapascals (MPa) with a subtle arc of variation (Figure 11①). The medial plateau exhibited the largest force distribution area at 0°, decreasing gradually from 3° to 18°. The peak VMS increased by 2.6 times, ranging from 4.13 to 14.05 MPa. The peak VMS remained relatively stable at 6°, 9°, and 12°, with a subsequent increase in the trend observed at 15° and 18° (Figure 11②). In contrast, the force distribution area of the lateral plateau was largest at 0° and gradually decreased from 3° to 18°, showing a nonsignificant trend of change. The peak VMS was highest at 0°, followed by a gradual decrease, with the peak VMS remaining relatively constant from 3° to 18° (Figure 11③).

**Figure 12.**
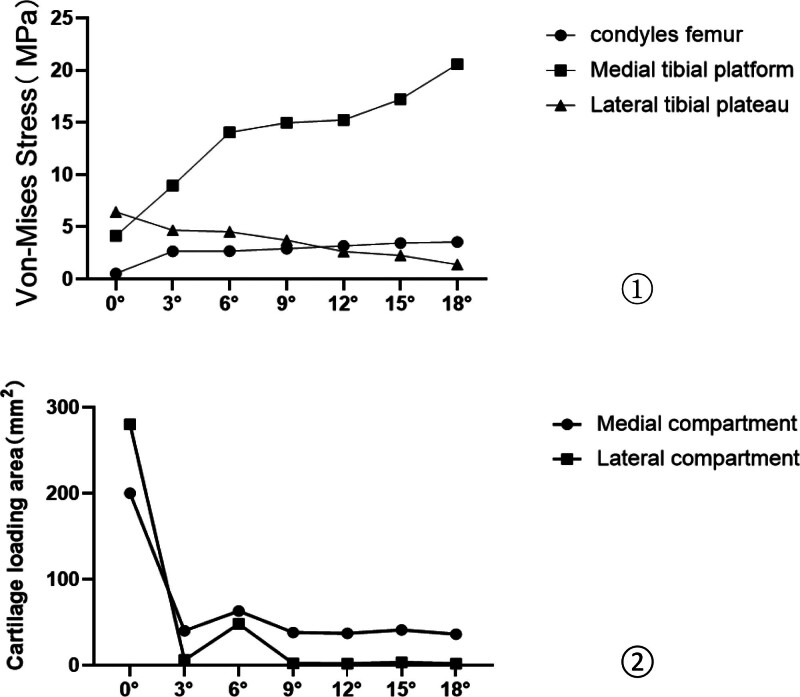
Distribution of peak VMS and area of cartilage loading area. ① Distribution of peak VMS of the cartilage of femoral condyle, medial plateau and lateral plateau; ② area of the cartilage loading area of medial compartment and lateral compartment. VMS = Von-Mises stress.

The cartilage loading area in the knee joint’s medial and lateral compartments exhibited its most significant change within the loading zone from 0° to 3°, with negligible alterations beyond 3° (Figure 12②), and there may have been a transition from faceted to point contact on the articular surfaces. Herein, larger load-bearing surfaces corresponded to reduced local loading. The peak VMS in the medial plateau was higher than that in the femoral condyles and lateral plateau when the varus angle exceeded 3°. With the gradual increase in varus angle, the peak VMS area of the femoral condyle shifted towards the medial condyle, while the peak VMS area of the medial plateau remained stable on the anteromedial side. These findings were consistent with clinical observations that cartilage damage in KOA with varus deformity tends to occur on the anteromedial side of the medial plateau.

### 4.2. Cartilage damage in KOA patients with different clinical valgus deformities of results

The cartilage damage in KOA patients with varying clinical valgus deformities was observed as follows: the finite element modeling groups based on varus deformity angles (0°, 3°, 6°, 9°, 12°, 15°, 18°) were correlated with corresponding clinical deformity angle groups (<3°, <6°, <9°, <12°, <15°, <18°, and ≥18°), and arthroscopic observations were made during surgery (Table 3). Significant correlations were identified between each grouping, with *P* < .01.

**Table 3:** Deformity angle and cartilage damage.

	*N*	*M*	*F*	*P*	Partial Eta Square
Knee joint cartilage	203	2.09	52.287	<.001	0.709
Medial plateau cartilage	203	3.23	312.679	<.001	0.729
Patellofemoral cartilage	203	2.66	55.486	<.001	0.691
Lateral plateau cartilage	203	1.25	15.335	<.001	0.581

*N* is the sample size, *M* is the mean, *F* is the ANOVA statistic, *P* is the level of significance, partial Eta Square, i.e., *η*^2^ is the estimated effect size.

**Figure 13.**
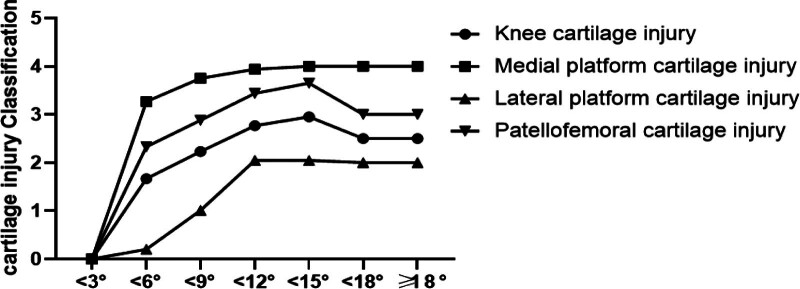
Line graph of the degree of cartilage damage in each compartment of the knee joint cavity for different deformity subgroups.

The clinical cases were predominantly within the varus deformity range of <9° and <12° (range, 6–11.9°), with cartilage damage in the medial plateau reaching grade 4 in this range. When the deformity angle was within the range of <6°, <9°, <12°, and <15° (range, 3–14.9°), joint damage peaked, and damage decreased in the group with varus angles of <18° and ≥18° (exceeding 15°). The cartilage damage in the medial plateau within the range of <9°, <12°, <15°, <18°, and ≥18° (exceeding 6°) remained relatively stable. The degree of damage in the patellofemoral articular space was higher than that in the lateral plateau (Table 4). The probability of cartilage damage increased when the varus angle was ≥6°, while joint damage decreased when the varus angle exceeded 15° (Fig. 13). It is worth noting that due to the reduced clinical sample size, the possibility of other injuries or patients being unable to bear weight while walking could not be excluded, leading to reduced joint weight-bearing.

**Table 4:** Degree of cartilage damage in each compartment of the knee joint cavity in different deformity subgroups.

	<3°	<6°	<9°	<12°	<15°	<18°	≥18°
Quantities	31	15	69	64	20	2	2
Knee joint cartilage	0.00	1.67	2.23	2.77	2.95	2.50	2.50
Medial plateau cartilage	0.00	3.27	3.75	3.94	4.00	4.00	4.0
Patellofemoral cartilage	0.00	2.33	2.88	3.44	3.65	3.00	3.00
Lateral plateau cartilage	0.00	0.20	1.01	2.05	2.05	2.00	2.00

## 5. Discussion

Knee valgus and varus deformities, which alter the mechanical axis of the lower limb, are significant contributing factors to the development of KOA, which often manifests as joint space narrowing and subchondral osteosclerosis, primarily resulting from an imbalance in stress distribution between the medial and lateral tibia.^[27]^ Lower limb varus deformity directly impacts the load-bearing capacity of the knee joint’s medial compartment, leading to increased pressure on the cartilage and subchondral bone. The imbalanced mechanical stress ultimately results in medial cartilage injury, which, in turn, accelerates the shift in the lower limb’s mechanical axis after medial plateau injury. This exacerbates force changes in other interarticular compartments, ultimately leading to cartilage damage in these compartments and further worsening KOA progression. Consequently, this shift in force distribution has prompted in vitro investigations into stress distribution and loading conditions in knee joint compartments.^[11,27,28]^ However, there has been limited analysis of in vitro stress distribution and loading conditions in conjunction with actual cartilage damage observed in clinical patients corresponding to specific deformities.

In our study, we employed a combination of CAD and finite element analysis to explore force distribution and loading in different varus angles and joint compartments under static conditions. This analysis was supplemented with data on cartilage damage observed in corresponding clinical patients. Our findings revealed that as the varus angle increases, there is a corresponding increase in the peak VMS in the medial plateau, with the VMS in this region significantly surpassing that of other compartments. Existing research indicates that moments generated during knee joint varus in the sagittal plane significantly increase, irrespective of active or passive movements. This elevation in VMS across compartments plays a crucial role in various functions, including joint stability and injury prevention,^[29]^ aligning with our study’s outcomes. Furthermore, cartilage damage exhibits a strong correlation with both peak VMS and the area under the peak. A wealth of research data underscores that in varus deformity, the majority of cartilage wear occurs in the anterior part of the medial plateau and the posterior part of the lateral plateau,^[30]^ consistent with the VMS peak area we have investigated.

The loss of plane motion in the knee is a critical factor impacting knee performance and susceptibility to injury.^[31]^ In particular, excessive sagittal plane motion has been proposed as a mechanism for lower extremity injuries, especially for knee injuries.^[32]^ Our clinical study results revealed that cartilage damage in the medial plateau began to manifest at valgus angles of 3° to 6°, with the peak VMS at 6° being nearly 3 times higher than at 0°. Between 6° and 15° of valgus, cartilage damage appeared in other compartments, suggesting that when the varus angle reaches 6°, cartilage damage in the knee exceeds its tolerance limit, which may not be limited to the sagittal plane alone but could also involve coronal plane movement, potentially accelerating intercompartmental injuries. As the knee valgus angle increases, the speed of joint movement in the sagittal plane also rises. This increased velocity can disrupt sagittal plane control, which has been proposed as a possible contributor to joint injury.^[33]^ Clinical study data further indicated that cartilage damage extended to the medial plateau, lateral plateau, and patellofemoral intercompartment within the 6° to 12° range, which infer that varus deformity initially affects the medial cartilage before spreading to other compartments. Once damage occurs in all 3 compartments, both the internal and external joint spaces become narrower, leading to increased bone volume and a decrease in the varus deformity angle. Ultimately, this compensatory mechanism contributes to the preservation of knee joint stability.

Furthermore, our clinical study results revealed a significant decrease in cases with valgus angles exceeding 15°. The lateral plateau exhibited varying degrees of injury exacerbation, while the peak VMS of the medial plateau displayed a slight arc increase, suggesting that abnormal knee joint activities may occur when the valgus angle reaches or exceeds 15°, disrupting sagittal plane balance and impeding the normal trajectory of joint movement. It cannot be ruled out that ligament injuries may result in joint instability and impaired normal ambulation. A valgus angle of 15° may represent a threshold beyond which varus deformity occurs. Relevant literature studies support this notion, indicating that as varus deformity worsens beyond 13° to 17°, wear is more likely to affect the central medial and posterior medial regions of the tibial plateau.^[34]^ Additionally, posterior medial tibial wear is associated with anterior cruciate ligament(ACL) injuries and excessive varus deformity, with a valgus angle of 15.5° serving as a threshold for identifying posterior medial tibial wear and 14.5° as a threshold for detecting ACL deficiencies.^[35]^ These findings align closely with our research outcomes.

Both clinical and in vitro biomechanical studies support the efficacy of restoring the mechanical axis to alleviate osteoarthritis.^[36]^ Biomechanical assessments can be performed using the finite element method, a computer-based approach for modeling knee joint mechanics.^[37]^ In our study, CAD software was utilized to simulate varus deformities for grouping and constructing 3-dimensional models, offering enhanced precision compared to 2-dimensional analyses.^[38]^ This approach allows for the simulation of multi-angle sagittal plane motion in the knee joint. Through the combination of CAD with Mimics software, we conducted various 3D image manipulations, such as scaling, free motion, and rotation, resulting in more accurate and intuitive varus modeling. The integration of CAD into our research streamlined the creation of precise finite element grouping models for cartilage loading under different varus deformities.^[13]^ Furthermore, CAD, in conjunction with Mimics software, facilitated varus angle measurements in each patient, reducing errors associated with previous 2D X-ray techniques and bringing the in vitro model closer to clinical reality. Consequently, we can offer clinical guidance regarding the necessary varus angle correction to modify articular cartilage loading and establish an optimal environment for cartilage repair.

### 5.1. Limitations

There were several limitations in our study. First, our finite element analysis only considered static cartilage loading on the weight-bearing surface in a straight position without simulating the dynamic motions of the human body or accounting for coronal plane movements. Clinical activities involve dynamic processes with various flexion and extension angles, impacting not only the weight-bearing surface but also the coronal surface. Furthermore, the influence of surrounding soft tissue moments on altering cartilage loading within each interarticular compartment was not considered.^[39]^ Additionally, besides the prevalent cartilage injuries observed in the medial plateau, we also frequently observed cartilage injuries in the patellofemoral compartment in clinical practice, which can be attributed to the involvement of the patella and surrounding muscles in the deformed medial knee, leading to overloading of cartilage in the patellofemoral compartment.

To address these limitations, future knee joint models could integrate factors related to muscles and ligaments while emphasizing the influence of patellar motion during various joint activities. It is also important to consider different body weights and diverse postures during motion to accurately replicate real knee joint movement states.^[40,41]^ Therefore, our future research would aim to identify multiple variables, focusing on factors like patellofemoral movement, variations in body weight and different sports postures to comprehensively investigate the loading conditions within interarticular compartments.

## 6. Conclusions

In summary, our study used a static finite element model to simulate varying cartilage loads in different compartments of the knee joint affected by varus deformity. By analyzing cartilage damage in conjunction with dynamic clinical cases, we established correlations between cartilage loads and damage in KV deformity. Our findings revealed a direct relationship indicating that as the varus deformity angle increases, the medial cartilage stress load also increases, leading to more pronounced cartilage damage in the corresponding peak stress area. Additionally, we observed coronal interpatellofemoral compartment cartilage damage when varus deformity exceeded 6°. Furthermore, varus angles exceeding 15° resulted in abnormal knee joint movements, and the probability of cartilage damage significantly increased with varus angles ≥6°, highlighting the importance of early surgical correction to prevent deformity exacerbation and restore optimal knee function.

## Author contributions

**Data curation:** Hongjie Zhang.

**Formal analysis:** Hongjie Zhang.

**Investigation:** Hongjie Zhang.

**Methodology:** Hongjie Zhang.

**Project administration:** Hongjie Zhang.

**Visualization:** Hongjie Zhang.

**Writing – original draft:** Hongjie Zhang.

**Writing – review & editing:** Hongjie Zhang.

**Conceptualization:** Jianxiong Ma, Aixian Tian, Haohao Bai, Jing Dai, Bin Lu.

**Software:** Yanfei Wu, Jiahui Chen.

**Funding acquisition:** Xinlong Ma.

**Resources:** Wei Luo.
